# Impact of applying a diabetes risk score in primary care on change in physical activity: a pragmatic cluster randomised trial

**DOI:** 10.1007/s00592-022-01895-y

**Published:** 2022-05-13

**Authors:** Esther Seidel-Jacobs, Fiona Kohl, Miguel Tamayo, Joachim Rosenbauer, Matthias B. Schulze, Oliver Kuss, Wolfgang Rathmann

**Affiliations:** 1 Institute for Biometrics and Epidemiology, German Diabetes Center (DDZ), Leibniz Center for Diabetes Research at Heinrich Heine University Düsseldorf, Auf’m Hennekamp 65, 40225 Düsseldorf, Germany; 2 German Center for Diabetes Research (DZD), 85764 Munich-Neuherberg, Germany; 3 Institute for Occupational, Social and Environmental Medicine, Centre for Health and Society, Faculty of Medicine, Heinrich Heine University Düsseldorf, Düsseldorf, Germany; 4 The Association of Statutory Health Insurance Physicians North Rhine, 40474 Düsseldorf, Germany; 5 Department of Molecular Epidemiology, German Institute of Human Nutrition Potsdam-Rehbruecke, 14558 Nuthetal, Germany; 6 Institute of Nutritional Science, University of Potsdam, Potsdam, Germany; 7 Centre for Health and Society, Medical Faculty and University Hospital Düsseldorf, Heinrich Heine University Düsseldorf, Düsseldorf, Germany

**Keywords:** Risk score, Risk prediction model, Type 2 diabetes, Prevention, Physical activity, Primary care

## Abstract

**Aim:**

There is little evidence of the impact of diabetes risk scores on individual diabetes risk factors, motivation for behaviour changes and mental health. The aim of this study was to investigate the effect of applying a noninvasive diabetes risk score in primary care as component of routine health checks on physical activity and secondary outcomes.

**Methods:**

Cluster randomised trial, in which primary care physicians (PCPs), randomised (1:1) by minimisation, enrolled participants with statutory health insurance without known diabetes, ≥ 35 years of age with a body mass index ≥ 27.0 kg/m^2^. The German Diabetes Risk Score was applied as add-on to the standard routine health check, conducted in the controls. Primary outcome was the difference in participants’ physical activity (International Physical Activity Questionnaire) after 12 months. Secondary outcomes included body mass index, perceived health, anxiety, depression, and motivation for lifestyle change. Analysis was by intention-to-treat principle using mixed models.

**Results:**

36 PCPs were randomised; remaining 30 PCPs (intervention: *n* = 16; control: *n* = 14) recruited 315 participants (intervention: *n* = 153; controls: *n* = 162). A slight increase in physical activity was observed in the intervention group with an adjusted mean change of 388 (95% confidence interval: − 235; 1011) metabolic equivalents minutes per week. There were no relevant changes in secondary outcomes.

**Conclusions:**

The application of a noninvasive diabetes risk score alone is not effective in promoting physical activity in primary care.

*Clinical Trial Registration*: ClinicalTrials.gov (NCT03234322, registration date: July 31, 2017).

**Supplementary Information:**

The online version contains supplementary material available at 10.1007/s00592-022-01895-y.

## Introduction

Early detection of people at risk for type 2 diabetes is necessary for effective prevention programs to reduce diabetes incidence [1]. Large clinical trials showed that physical activity, healthy dietary patterns and weight loss reduce diabetes risk by > 50% [1]. For identification of high-risk individuals for diabetes, primary care physicians (PCPs) play a central role, because they are the first point of contact for diabetes-related issues. Routine health checks, offered by PCPs, are one opportunity for early detection of diabetes and associated risk factors [2]. For the identification of persons at risk, diabetes risk scores alone or in combination with the measurement of blood glucose measures (fasting plasma glucose, oral glucose tolerance test, haemoglobin A1c (HbA1c)) can be used [3]. Diabetes risk scores include relevant risk factors aiming to estimate the probability for an individual to develop diabetes within a defined time span [4]. In the last years, more than three hundred diabetes risk scores were developed worldwide, but only few have been externally validated and are available for routine use in clinical practice [5–8].

Diabetes prevention and care guidelines worldwide have included diabetes risk scores as adequate tools for identifying people at risk [3, 9, 10]. Despite the demands of many experts for more research on the practical application, little is known about the effectiveness of applying diabetes risk scores in primary care on individual risk factors, motivation for behaviour changes or if it has adverse psychological effects (e.g. depression) [5, 7]. This is in contrast to research on cardiovascular risk scores for primary prevention of cardiovascular diseases (CVD) [11, 12]. During the last 2 decades, numerous randomised controlled trials on the effectiveness of using cardiovascular risk scores showed that there is an insignificant influence on individual risk factors and a lack of effectiveness in reducing CVD events and mortality [11]. To add evidence of clinical effectiveness in diabetes research, the aim of the study was to investigate the impact of applying a noninvasive risk score in primary care as add-on of routine health checks on change in individuals’ physical activity and further health related outcomes.

## Methods

### Design and Study Participants

The study was a pragmatic blinded parallel group superiority cluster randomised controlled trial [13]. A cluster randomisation was chosen to avoid contamination effects between participants. Clusters were PCPs located in the region of the federal state of North Rhine-Westphalia in Germany. PCPs with and without further training in diabetology according to German Diabetes Association standards, who provided the routine health check (“Check-up 35”) to people with statutory health insurance (which have 89% of the general population) were invited to participate with written information, a consent form and baseline questions needed for randomisation. After consenting to participate in the study, PCPs were randomised into an intervention or control group using the minimization technique [14] with an 1:1 allocation ratio to balance for properties of PCPs by a blinded clinical data manager. The study personnel informed PCPs about group allocation and in a face-to-face meeting with each PCP, all study aspects were discussed. PCPs were constrained to enrol consecutively participants who fulfilled the following inclusion criteria: appointment for the routine health check, statutory health insurance, age ≥ 35 years, and body mass index (BMI) ≥ 27 kg/m^2^. People were not eligible if they have been diagnosed with type 1 or type 2 diabetes or had already at least one measurement of abnormal blood glucose level (fasting glucose ≥ 126 mg/dl [7 mmol/L] or 2 h oral glucose tolerance test ≥ 200 mg/dl [11 mmol/L] or HbA1c ≥ 6.5% [48 mmol/mol]) before the routine health check. All participants provided written consent at the day of inclusion. Participants and the statistician assessing the outcomes were blinded regarding the assignment to the intervention.

#### Control and intervention

In the control group, the routine health check was conducted, including the measurement of blood pressure, total cholesterol, fasting glucose, and urine test. At the end of the health check, PCPs provided a short non-standardized individual consultation.

In the intervention group, the German Diabetes Risk Score (GDRS) was used as add-on to the routine health check [4]. The validated GDRS is based on a prospective cohort study (European Prospective Investigation into Cancer and Nutrition [EPIC]-Potsdam study, *n* = 25,000) and provides good prediction of the 5-year risk of type 2 diabetes (receiver operating characteristic curve [95%-confidence interval; CI]: 0.87 [0.83; 0.90]) [15]. The GDRS focuses on important noninvasive risk factors to predict the 5-year diabetes risk. It contains a visual presentation of the individual diabetes risk with categorisation in risk groups and short recommendations to enhance a healthy lifestyle. The completed two pages GDRS by study participants during waiting times of the routine health check served as a practical guide for discussion of individual tailored preventive strategies at the end of the health check.

### Outcomes

The primary outcome of the study was the difference of participants’ physical activity between the intervention and control group at 12 months after the routine health check. We used the International Physical Activity Questionnaire Short Last 7 Days Format (IPAQ-SF) [16] and calculated the total metabolic equivalent of task minutes per week (MET-min/week) of vigorous and moderate physical activity and walking over the past 7 days according to the IPAQ manual. Additionally, a categorical variable with three levels of physical activity was calculated [16].

Secondary outcomes were the difference between the intervention and control group at 12 months follow-up in:

- *BMI (kg/m*^*2*^*) and waist circumference (WC) (cm)* At baseline, the PCP or medical assistant objectively measured weight, height and WC and simultaneously trained participants to measure WC themselves. For weight and WC measurement during follow-up, an instruction manual was provided and every participant received a measuring tape for home use.
- *Perceived health* Assessed by the overall question “How is your health in general?“, with five answer alternatives from “very good” to “very bad” (1–5 points) recommended by World Health Organization assessed perceived health [17]. Higher values indicate a poorer rating of health.
- *Levels of depression and anxiety* Assessed by the Hospital Anxiety and Depression Scale German Version (HADS-D) was used, which is a validated 14 items questionnaire showing good reliability and validity in the general population [18]. For depression and anxiety, two scores are given (range 0–21 points) for which higher scores indicate higher depression or anxiety disorder (< 8: normal, 8–9: borderline, > 10: mood disorder).
- *The motivation to change lifestyle* The transtheoretical stage of change model (SOC) measured motivation to change to higher physical activity level, healthy diet, reduce body weight and quit smoking [19]. Participants were classified into a stage (Precontemplation, Contemplation, Preparation, Action or Maintenance) by a defined algorithm derived from previous studies (Supplementary Table 1) [19, 20].

### Statistical analysis

Sample size calculation, which lead to a sample size of 300 participants, split up on 15 PCPs per study group, has been described [13]. Analysis was conducted according to intention-to-treat principle. For analysis of all outcomes, linear (for continuous outcomes) or generalized linear with a logit link (for binary outcomes) mixed models were estimated including the intervention effect, a random intercept to adjust for the cluster effect, the respective baseline value, and covariates age, sex and smoking status of participants at baseline. Furthermore, the variables of PCPs used for minimization, sex, medical specialization of PCP (internal medicine/ general medicine/ medical practitioner) and further training in diabetology (yes/no) according to the German Diabetes Society and the socio-economic environment of the practices, based on statistical data about unemployment rates in the defined region were included as covariates. For assessment of changes in motivation to change lifestyle (SOC), the stages action and maintenance were combined. Analysis of motivation change in physical activity was conducted without people having a physical limitation at follow-up, and motivation to quit smoking excluded participants who were never smoker at baseline.

Three sensitivity analyses were performed:

(I) A statistical analysis was conducted in which missing binary and continuous variables were imputed by multiple across-cluster imputation. (II) Because the follow-up of the study coincided with the SARS-CoV-2 pandemic, we investigated whether times of hard lockdown (March 2020 to June 2020 and December 2020 to February 2021) due to Covid-19 had an impact on the change in the primary outcome. (III) The difference of participants’ physical activity between the intervention and control group at 12 months after the routine health check was assessed only in those with low physical activity at baseline. For these two analyses, an interaction term of intervention group and time point or physical activity (in three categories: low, moderate and high physical activity) was included in the mixed model for the primary outcome.

Ethical approval was received from the ethics committee of the Heinrich Heine University Düsseldorf in June 2017 (Reference-No: 5540). All participants provided written informed consent. The trial is registered on ClinicalTrials.gov (NCT03234322, registration date: July 31, 2017). Study data were collected and managed using REDCap electronic data capture tools hosted at the German Diabetes Center (DDZ) [21]. The analyses were carried out using SAS version 9.4 (SAS Institute, Cary, NC).

## Results

**Fig. 1:**
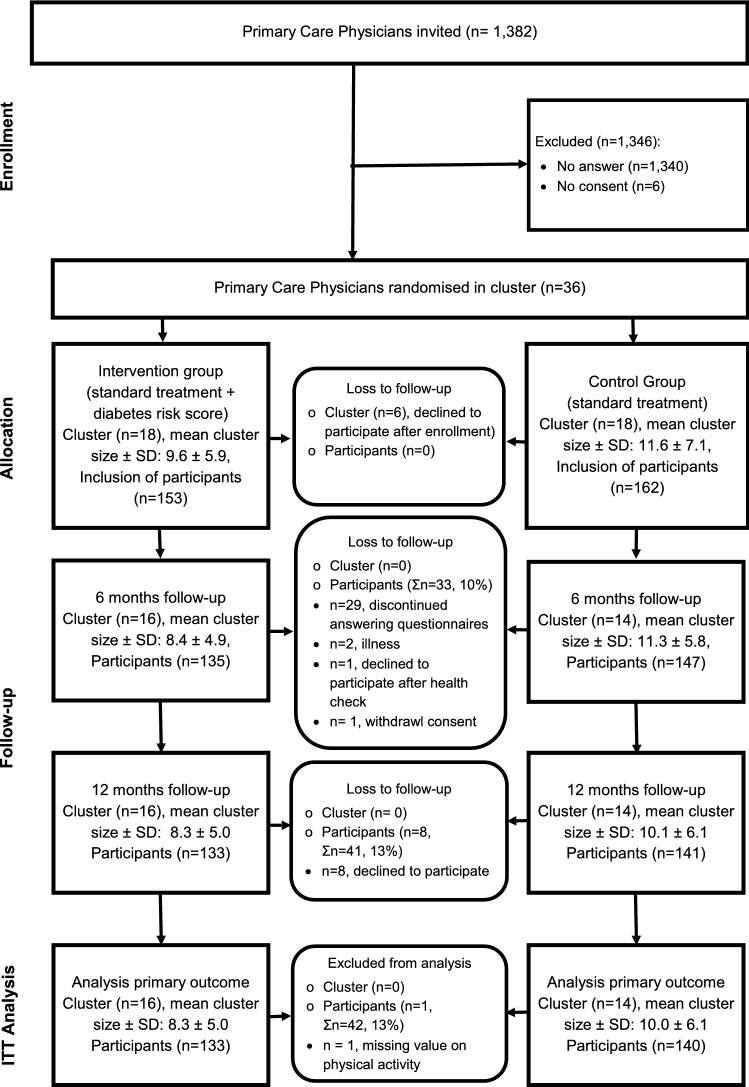
Flow-chart

Between July 2017 and September 2018, 36 PCPs consented to participate and were randomised to the intervention or control group (Fig. 1). Of those, six PCPs declined to participate early after enrolment and did not recruit any participants. The remaining 30 PCPs included 315 participants (intervention: *n* = 153; control group: *n* = 162) until January 2020. At the end of the study (February 2021), 274 of 315 (87%) included participants completed the follow-up.

### Sample characteristics

Baseline characteristics of the PCP and participants were similar in the intervention and control group (Table 1). Baseline characteristics of completers and non-completers of 12-month follow-up are presented in Supplementary Table 2.

**Table 1:** Baseline characteristics of primary care physicians and participants by study group

	Intervention group	Control group
*Primary care physicians’ characteristics*	*16*	*14*
Sex, men	8 (50)	9 (64)
Age, years	54 ± 8.1	49 ± 8.0
Years since residency	14.4 ± 6.8	12.2 ± 9.7
Primary care physicians’ specialization		
General practicioner	5 (31)	6 (43)
Medical practitioners without specialisation	1 (5)	0 (0)
Internists	10 (63)	8 (57)
Diabetologist DDG^a^	3 (19)	0 (0)
Socio-economic environment of the practices		
Low to medium	5 (31)	3 (21)
Medium	4 (25)	2 (14)
High	7 (44)	9 (64)
Patient contacts per week	140 (105; 190)	145 (120; 168)
Routine health checks per quarter	88.3 ± 51.7	119.3 ± 35.9
Percentage of privately insured patients	16.8 ± 6.0	11.8 ± 7.9
Percentage of patients over 65 years	40.8 ± 16.9	47.0 ± 15.5
Percentage of extreme obese patients (BMI ≥ 35 kg/m^2^)	12.5 (5; 25)	20.0 (15; 25)
*Participants’ characteristics*	*153*	*162*
Sex, men	61 (40)	88 (54)
Age, years	55 ± 11.5	57 ± 11.4
German nationality	141 (92)	149 (92)
Education		
Less than high school	88 (58)	90 (56)
High school	40 (26)	44 (27)
College/university	25 (16)	28 (17)
Occupation		
Full/part time (≥ 15 h/week)	101 (66)	98 (61)
Marginally (< 15 h/week)	5 (3)	5 (3)
Unemployed	7 (5)	11 (7)
Retired	35 (23)	43 (27)
Other	5 (3)	5 (3)
Physical activity (IPAQ-SF MET-min/week)	2080 (954; 3810)	2070 (918; 4074)
Low physical activity	43 (28)	47 (29)
Moderate physical activity	62 (40)	55 (34)
High physical activity	48 (31)	60 (37)
5-year diabetes risk (GDRS-Score)	60 ± 12.6	–
Low risk (< 46 points)	23 (15)	–
Still low risk (46–56 points)	31 (20)	–
Elevated risk (57–63 points)	33 (22)	–
High to very high risk (> 63 points)	66 (43)	–
Smoking status		
Smoker	38 (25)	33 (20)
Ex-smoker^b^	63 (41)	66 (41)
Never smoker	52 (34)	63 (39)
History of cardiovascular disease^c^	6 (4)	15 (9)
Diagnosed hypertension (> 140/90 mmHg)	72 (47)	91 (57)
Total cholesterol mmol/L	5.3 ± 1.1	5.5 ± 1.2
HDL cholesterol mmol/L^d^	1.4 ± 0.4	1.3 ± 0.4
Triglycerides mmol/L^d^	1.4 (1.9; 1.8)	1.5 (1.0; 2.2)
Fasting plasma glucose mg/dL [mmol/L]	89.4 ± 15.3 [5.0 ± 0.9]	88.1 ± 16.1 [4.9 ± 0.9]
Random glucose mg/dl [mmol/L]^e^	101.5 (89.0; 114.0) [5.6 (4.9; 6.3)]	91.0 (80.0;112.0) [5.1 (4.4; 6.2)]
HbA1c (% [mmol/mol])^d^	5,6 ± 0.4 [37.9 ± 4.8]	5.7 ± 0.4 [38.8 ± 3.9]
Glucose disorder status^f^		
No diabetes	108 (71)	117 (72)
Prediabetes	42 (28)	42 (26)
Suspicion of diabetes	3 (2)	3 (2)

Data are n (%), mean ± SD, or median (25th; 75th percentile)

*DDG* German diabetes society, *GDRS* German diabetes risk score, *HADS-D* The hospital anxiety and depression scale German version, *HbA1c* haemoglobin A1c, *HDL* high-density lipoprotein, *IPAQ-SF* physical activity questionnaire short last 7 days format, *MET* metabolic equivalent of task

^a^Further education according to the German Diabetes Society guidelines

^b^Ex-smoker for more than 6 months

^c^At least one diagnosis: coronary heart disease, peripheral arterial disease or stroke

^d^More than 10% missing values, not part of routine check-up, voluntary information from the PCP

^e^Participant not fasting before glucose testing, n = 12

^f^According to diabetes diagnosis criteria, ADA Guideline

### Main results

**Fig. 2:**
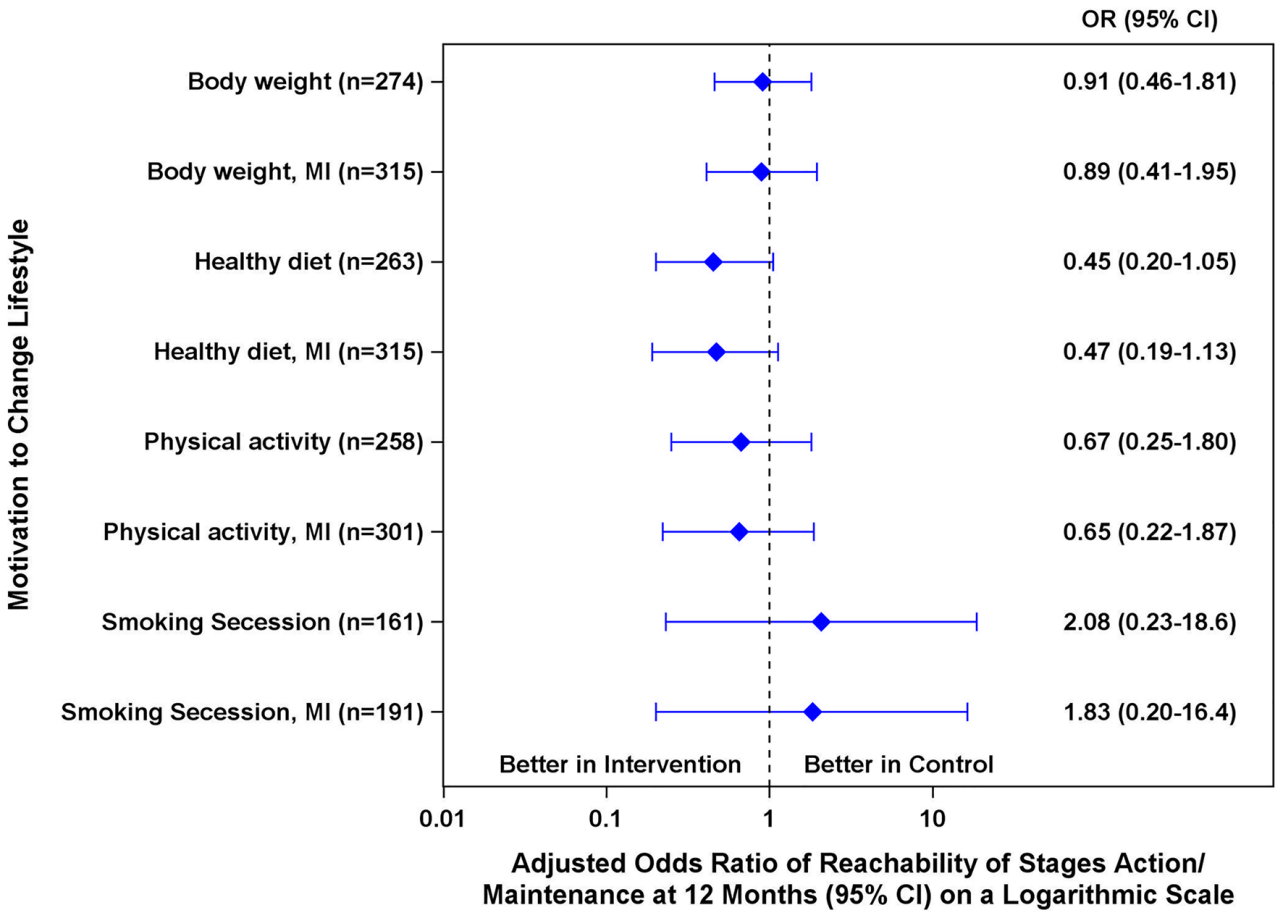
Odds ratio (OR) on reachability of stages action or maintenance at 12 months follow-up. Results labelled with MI refer to results after multiple across-cluster imputation of missing values

Raw data (mean ± SD, or median [25th; 75th percentile] of continuous outcomes by time point of the study (baseline, 6 and 12 month follow-up), as well as results of mixed models (according to the intention to treat principle and after multiple imputation as sensitivity analysis) with corresponding ICCs are presented in Table 2. Results of categorical outcomes on motivation to change lifestyle are presented in Fig. 2 and Supplementary Table 3.

**Table 2:** Primary and secondary continuous outcomes and adjusted mean group difference after 12 months follow-up

Outcome	Intervention group			Control group			Β (95% CI)^a^	Β (95% CI) MI^a^	ICC (95% CI)
	Baseline	6 month follow-up	12 month follow-up	Baseline	6 month follow-up	12 month follow-up			
Physical activity (IPAQ-SF) MET-min/week	2080 (954; 3810)	2280 (1070; 4570)	2730 (990; 4520)	2070 (918; 4074)	2130 (792; 4360)	2080 (975; 4100)	388 (− 235; 1011)	362 (− 285; 1009)	0.00 (–;–)
BMI (kg/m^2^)	32.8 ± 4.86	32.1 ± 5.02	32.1 ± 5.12	32.5 ± 5.12	32.0 ± 4.97	32.0 ± 4.65	− 0.27 (− 0.91; 0.37)	− 0.23 (− 0.93; 0.46)	0.10 (0.02; 0.18)
Waist circumference (cm)	109 ± 12.5	107 ± 11.6	107 ± 12.7	110 ± 13.8	109 ± 15.7	107 ± 11.6	0.61 (− 1.35; 2.58)	1.32 (− 1.68; 4.31)	0.00 (0.00; 0.01)
Perceived health	2.38 ± 0.70	2.35 ± 0.74	2.33 ± 0.69	2.42 ± 0.70	2.37 ± 0.67	2.42 ± 0.67	− 0.09 (− 0.26; 0.08)	− 0.06 (− 0.20; 0.08)	0.03 (− 0.02; 0.08)
Level of anxiety (HADS-D)	6.07 ± 3.93	5.58 ± 3.93	5.32 ± 3.78	5.73 ± 4.01	5.14 ± 3.98	5.17 ± 3.72	0.47 (− 0.35; 1.29)	0.44 (− 0.46; 1.34)	0.05 (− 0.02; 0.11)
Level of depression (HADS-D)	5.00 ± 3.61	4.38 ± 3.86	4.25 ± 3.79	4.51 ± 3.66	4.38 ± 3.74	4.43 ± 3.87	− 0.07 (− 0.70; 0.56)	− 0.10 (− 0.77; 0.57)	0.00 (–;–)

Data are n (%), mean ± SD, or median (25th; 75th percentile)

*HADS-D* The hospital anxiety and depression scale German version, *ICC* intracluster correlation coefficient, *IPAQ-SF* international physical activity questionnaire short last 7 days format, *MET* metabolic equivalent of task, *MI* mixed models conducted after across cluster multiple imputation of missing values (sensitivity analysis)

^a^Estimated from mixed models for each outcome including a random intercept to adjust for cluster effect, the respective baseline value of the outcome, and the covariates age, sex and smoking status of participants at baseline, and the variables used for minimization [sex and medical specialization of PCP, further training in diabetology and the socio-economic environment of the practices]

#### Primary outcome

Physical activity increased during 12 months follow-up in the intervention group (+ 650 MET-min/week), whereas no change was observed in the control group (+ 10 MET-min/week). The mixed model showed an adjusted mean group difference of 388 MET-min/week (95% CI − 235; 1010).

#### Secondary outcomes

BMI and WC slightly decreased in both groups during follow-up. The mean group difference was − 0.3 (95% CI − 0.9; 0.4) kg/m^2^ in BMI and − 0.6 (95% CI − 1.4; 2.6) cm in WC after 12 month follow-up.

Perceived health was constant at 2.3 and 2.4 points in intervention and controls group, which indicates good to fair self-rated health. In the mixed model, the adjusted difference in means was small (− 0.1; 95% CI − 0.3; 0.1 points).

The mean score levels of anxiety and depression were below eight in both groups and at all time points. This indicates no mood disorders in the majority with only minimal adjusted differences in means between the groups at 12 month follow-up (0.5; 95% CI − 0.4; 1.3 in anxiety and 0.1; 95% CI − 0.7; 0.6 in depression score).

The odds to sustainably change to a healthy lifestyle (measured by reaching motivation stages action/maintenance) were reduced in the control group compared to the intervention group by 9% (Odds Ratio (OR) 0.91, 95% CI 0.46; 1.81) for body weight, 33% (OR 0.67, 95% CI 0.25; 1.80) for physical activity, and 55% (OR 0.45, 95% CI 0.20; 1.05) for healthy diet. Regarding smoking cessation, the odds more than doubled in the control compared to the intervention group, however, with a large CI (OR 2.08, 95% CI 0.23; 18.6).

### Sensitivity analyses

(I) Multiple across-cluster imputation did not change the results remarkably (Table 2, Fig. 2). (II) Analysis of the impact of the Covid-19 lockdown on physical activity showed that those in the intervention group were more active in lockdown than in no-lockdown times with a difference of more than 1300 MET-min/week (control group difference = 139 MET-min/week) (Supplementary Fig. 1). (III) The difference between the intervention and control group at 12 months follow-up of those with low physical activity at baseline showed a small difference of 305 MET-min/week with a large CI (95% CI − 820; 1430). In the intervention group, 43 (28%) and in the control group 47 (29%) participants had a low physical activity at baseline.

## Discussion

This study showed that only applying a diabetes risk score with a subsequent discussion of results as part of a routine health check is not sufficient to promote physical activity. Only a slight increase in physical activity after 12 months of 388 MET-min/week was observed, which is of limited clinical relevance. In addition, changes in BMI and WC were minimal between the intervention and control groups. Constant levels of health, anxiety and depression also showed the small effect of the intervention. The odds to sustainably change to a healthy lifestyle (reduce body weight, increase physical activity and healthy nutrition) was somewhat increased in the intervention group.

Up to now, only few diabetes risk scores have been used as part of an impact study to change patient outcomes [5]. Comparable to the current study, a randomised controlled trial with healthy middle-aged adults also did not find a sufficient effect of a risk score on physical activity after provision of a genetic or phenotypic risk score assessment of type 2 diabetes in addition to standard lifestyle advice [22]. After 8 weeks, the mean difference in physical activity between the phenotypic risk group and control group was 1.3 kJ/kg/d [95% CI − 1.6; 4.3] which was smaller than the minimal clinically relevant effect as defined before the study. Moreover, differences in weight [− 0.3 kg (95% CI − 0.1; 0.3)], and behaviour change on lifestyle habits [e.g. intention to change physical activity: − 0.1 point change (− 0.2; 0.1)] were small [22]. However, the study duration was short and the study was not conducted in the primary care setting. In addition, participants had a lower mean BMI than in our study (BMI: 26.1 vs. 32.6 kg/m^2^). No changes were also seen for self-rated health, worry and anxiety.

A different approach was used by a non-randomised study using electronic medical records from 455 general practices in England, in which effectiveness of a diabetes screening component in a national cardiovascular risk assessment program was evaluated [23]. Analysis of 387,000 individuals aged 40–74 years showed that general practices’ actively participating in the NHS Health Check programme using a diabetes risk score identified more people with non-diabetic hyperglycaemia and undetected type 2 diabetes. Additionally, those practices provided better glucose and cardiovascular risk management [23]. Those findings indicate that a minimal intervention like provision of a diabetes risk score as component of routine health checks may has some effects on patient management in primary care.

Comparable studies in the field of primary prevention of CVD in primary care have also indicated insufficient or minimal clinical effectiveness of cardiovascular risk scores alone [11, 12]. Umbrella reviews on this topic showed little or no effect on lifestyle behaviour and on psychological (e.g. anxiety, depression) or physical adverse effects [11, 12]. Moreover, inconclusive evidence was found on the change in cardiovascular risk and no improvement in patient-relevant outcomes (reduction of cardiovascular death or fatal and non-fatal cardiovascular events) [11]. However, the accuracy of risk perception increased and there was a slight reduction of blood pressure, total cholesterol and smoking levels, especially in high-risk patient groups [12].

Both for cardiovascular and diabetes risk, the mode of medical counselling used by PCPs is important. An umbrella review showed that counselling of physical activity is an important aspect of primary diabetes prevention especially when multiple behavioural change techniques are used, e.g. fix targets and written prescriptions [24]. Therefore, the application of a diabetes risk score in combination with an appropriate counselling on physical activity and other health related aspects (smoking, weight reduction) may lead to clinical significant increases in physical activity.

### Strengths and limitations

A main strength of the study is the cluster randomised trial design with well-balanced characteristics of PCPs and participants. Using self-reported measures for physical activity is a limitation of the study. Validation studies found that the IPAQ-SF overestimates physical activity level [25]. However, studies on the IPAQ-SF has been consistently shown to have a high reliability (0.66–0.88) [25]. The focus of this study was on the individual change of physical activity and groups were compared according to the IPAQ-SF adjusting for the baseline value. Hence, possible overestimations of the IPAQ-SF instrument should be independent of the intervention. WC and weight were also self-reported by the participants in the follow-up. However, participants were trained to measure WC by the PCP or medical assistant and received an instruction manual and measuring tape at baseline. Furthermore, it should be noted that in the GDRS, the positive effect of physical activity on diabetes risk is calculated to be small and predominately depicted over waist circumference. Therefore, it is possible that physical activity has been underestimated to reduce diabetes risk by PCPs and participants, although primary prevention studies have shown the positive effect of physical activity on diabetes risk. Moreover, most people in our study stated to be moderately active at baseline (40% and 34% in intervention and control group), which could also had an impact of our primary outcome. However, the group comparison of those with low physical activity at baseline showed also no clinical relevant differences between the intervention and control group (305 MET-min/week; 95% CI − 820; 1430). A reviewer wondered whether the results of our primary analysis would be replicated when using the familiar categorization of the outcome (low, moderate and high physical activity). We therefore fitted a proportional-odds model to the ordinal outcome. The fully adjusted proportional-odds model showed that in the intervention group, the odds of being in a higher physical activity group was 14% larger than in the control group (odds ratio: 1.14; 95% CI 0.67; 1.95). As expected and conforming the analysis for the continuous version of the outcome, this result is neither statistically significant nor clinically relevant. An additional limitation is that the follow-up of the study coincided with the SARS-CoV-2 pandemic including times of hard lockdown, which could have had an impact especially on physical activity. The sensitivity analysis revealed an increase in physical activity during times of lockdown. Systematic reviews showed that most studies found a reduction of physical activity [26, 27]. Interestingly, two studies showed that highly active people as well as physical activity beginners maintained or increased their physical activity level, which corresponds with our results [26]. However, it should be considered, that a majority of these studies used non-validated physical activity questionnaires and the sample size included in the sensitivity analysis of our study was small, because only 35 people (13%) answered the last questionnaire in times of hard lockdown. Lastly, it should be mentioned, that the Federal Joint Committee decided some amendments of the routine health check during the study. From April 2019, changes included a deeper focus on individual cardiovascular risk factors (and if indicated using a cardiovascular risk score) and a risk-adapted counselling. This change has the potential for a slight dilution of the intervention effect. However, there was a transitional period of some months, leaving only few months of study time with some participants, thus, we are convinced that this did not have a relevant impact on the study results.

In conclusion, we found that the application of a diabetes risk score as part of a routine health check in primary care did only have minimal effects on important diabetes risk factors physical activity, and secondary outcomes. Overall, the available limited evidence underpins the importance of more randomised impact studies, which take into account important endpoints, e.g. morbidity and mortality, to confirm the effectiveness or lack of effectiveness of diabetes risk scores.

## Supplementary Information

Below is the link to the electronic supplementary material.

Supplementary file1 (PDF 85 kb)

Supplementary file2 (PDF 599 kb)

## Data Availability

The datasets analysed during the current study are available from the corresponding author on reasonable request.
