# Supplementary material for: Impact of applying a diabetes risk score in primary care on change in physical activity: a pragmatic cluster randomised trial
Source: Acta Diabetol. 2022 May 13;59(8):1031–40. doi: 10.1007/s00592-022-01895-y (PMC9098381; doi:10.1007/s00592-022-01895-y)

Supplementary Information

Title: Impact of Applying a Diabetes Risk Score in Primary Care on Change in Physical Activity - A Pragmatic Cluster Randomised Trial

Authors: Esther Seidel-Jacobs, Fiona Kohl, Miguel Tamayo, Joachim Rosenbauer, Matthias B. Schulze, Oliver Kuss, Wolfgang Rathmann

Journal: Acta Diabetologica

Corresponding address:

Dr. Esther Seidel-Jacobs, MPH

Institute for Biometrics und Epidemiology, German Diabetes Center (DDZ)

Leibniz Center for Diabetes Research at the Heinrich Heine University Düsseldorf

Auf'm Hennekamp 65

40225 Düsseldorf, Germany

Phone: (+49)211-3382-338

Fax: (+49)211-3382-677

esther.seidel-jacobs@ddz.de

Table 1: Definition of stages of the transtheoretical stage of change model (SOC)

Precontemplation	Participants are not considering changing behaviour in the next 6 months, are not intensively physical active on >20 minutes on three days per week, try not to eat little sugar or fat and little alcohol, lots of vegetables, fruit and whole grain products, try not to lose weight or are smokers.
Contemplation	Patients are considering a change in behaviour in the next 6 months.
Preparation[a]	Patients are seriously planning to change behaviour. They are confident that they will make changes within the next month.
Action	Patients have changed behaviour within the last 6 months.
Maintenance	Patients have maintained the change for at least 6 months

[a]Because of a slightly different structure of the questions to reduce body weight, the stage preparation is not defined (Rossi JS, Susan RR, Velicer WF, Prochaska JO. Motivational readiness to control weight. In: Allison DB, editor. Methods for the assessment of eating behaviours and weight related problems. Newbury Park, CA: Sage 1995. p. 381-424.)

Table 2: Baseline characteristics of participants who completed the follow-up compared with non-completers by study group

	Group							
	Intervention				Control			
**Variable**	**Analysed at 12 months (n=133)**		**Non-completers (n=20)**		**Analysed at 12 months (n=140)**		**Non-completers (n=22)**	
	N		N		N		N	
**Physical Activity**								
Continuously (IPAQ-SF MET-min/week)	133	2,120 (990; 4120)	20	1,680 (813; 2,510)	140	1,960 (834;4,070)	22	2,200 (1,310; 4,930)
Categorised (IPAQ-SF MET-min/week)	133		20		140		22	
low physical activity		38 (29)		5 (25)		43 (31)		4 (18)
moderate physical activity		49 (37)		13 (65)		45 (32)		10 (46)
high physical activity		46 (35)		2 (10)		52 (37)		8 (36)
**Basic characteristics**								
Sex, men	133	48 (36)	20	13 (65)	140	76 (54)	22	12 (55)
Age, years	133	55 ± 11.1	20	52 ± 13.9	140	56 ± 11.4	22	57 ± 12.0
Education	133		20		140		22	
Less than High School		76 (57)		12 (60)		77 (55)		13 (59)
High School		35 (26)		5 (25)		40 (29)		4 (18)
College/ University		22 (17)		4 (18)		23 (16)		5 (23)
**Health variables**								
5-year diabetes risk (GDRS-Score)	133	60.5 ± 12.7	20	58.3 ± 12.0	..	..	..	..
low risk (< 46 points)	133	20 (15)	20	3 (15)	..	..	..	..
still low risk (46-56 points)		25 (19)		6 (30)		..		..
elevated risk (57-63 points)		30 (23)		3 (15)		..		..
high to very high risk (> 63 points)		58 (44)		8 (40)		..		..
Body Mass Index (kg/m^2)	133	32.7 ± 5.0	20	33.2 ± 4.0	140	32.4 ± 5.0	22	31.4 ± 5.9
Waist circumference (cm)	133	109.2 ± 12.4	20	110.6 ± 13.0	138	109.6 ± 13.6	22	114.0 ± 14.3
Subjective Health	132		19		137		22	

Very good/ good		79 (60)		12 (63)		76 (56)		12 (55)
Moderate/bad/very bad		53 (40)		7 (37)		61 (45)		10 (46)
HADS-D present anxiety disorder	133	15 (11)	20	5 (25)	140	16 (11)	22	2 (9)
HADS-D present depression	133	15 (11)	20	1 (5)	140	14 (10)	22	2 (9)
Smoking status	133		20		140		22	
Smoker		31 (23)		7 (35)		25 (18)		8 (36)
Ex-smoker[a]		57 (43)		6 (30)		55 (39)		11 (50)
Never smoker		45 (34)		7 (35)		60 (43)		3 (14)
**Laboratory variables**								
History of cardiovascular disease[b]	133	4 (3)	20	2 (10)	140	14 (10)	22	1 (5)
Diagnosed Hypertension (>140/90 mmHg)	133	65 (49)	20	7 (35)	139	75 (54)	22	16 (73)
Fasting plasma glucose mg/dL [mmol/L]	132	89.2 ± 14.9 [5.0 ± 0.8]	19	90.5 ± 17.7 [5.0 ± 1.0]	134	87.7 ± 16.2 [4.9 ± 0.9]	21	90.9 ± 15.3 [5.1 ± 0.9]
Random glucose mg/dl [mmol/L][c]	1	114.0 ± ·· [6.3 ± ··]	1	89.0 ± ·· [4.9 ± ··]	8	94.8 ± ·· [5.3 ± ··]	2	91.0 ± ·· [5.1 ± ··]
HbA1c (%) [mmol/mol][d]	52	5.6 ± 0.4 [37.6 ± 4.8]	8	5.8 ± 0.4 [39.8 ± 4.7]	45	38.8 ± 4.0 [5.7 ± 0.4]	3	38.8 ± 0.0 [5.7 ± 0.0]
Glucose disorder status[e]	133		20		140		22	
No Diabetes		96 (72)		12 (60)		102 (73)		15 (68)
Prediabetes		35 (26)		7 (35)		35 (25)		7 (32)
Suspicion of diabetes		2 (2)		1 (5)		3 (2)		0 (0)

Data are n (%), mean ± SD, or median (25th; 75th percentile).

[a]Ex-smoker for more than 6 months; [b]At least one diagnosis: coronary heart disease, peripheral arterial disease or stroke; [c]Participant not fast before glucose testing; [d]More than 10% missing values, not part of routine health check, voluntary information from the PCP, n=12; [e]According to diabetes diagnosis criteria, ADA Guideline.

IPAQ-SF = Physical Activity Questionnaire Short Last 7 Days Format; MET = Metabolic equivalent of task; HADS-D = The Hospital Anxiety and Depression Scale German Version; HbA1c = Hemoglobin A1c test

Table 3: Adjusted odds ratio for impact of diabetes risk score on reachability of stages action/maintenance

		Intervention Group		Control Group		Control vs. Intervention Group		
**Behavior Change**	**Motivation Stage**	**Baseline**	**12 month follow-up**	**Baseline**	**12 month follow-up**	**Odds Ratio (95% CI)**	**Odds Ratio (95% CI) MI**	**ICC (95% CI)**
Body weight	Precontemplation/ Contemplation	120 (78.4)	94 (70.7)	131 (80.9)	103 (73.1)	0.91 (0.46; 1.81)	0.89 (0.41; 1.95)	0.03 (-0.03; 0.09)
	Action/ Maintanance	33 (21.6)	39 (29.3)	31 (19.1)	38 (27.9)			
Physical activity	Precontemplation/ Contemplation/ Preparation	92 (62.2)	68 (54.4)	97 (63.4)	83 (62.4)	0.67 (0.25; 1.80)	0.65 (0.22; 1.87)	0.17 (0.03; 0.31)
	Action/ Maintanance	56 (37.8)	57 (45.6)	56 (36.6)	50 (37.6)			
Healthy diet	Precontemplation/ Contemplation/ Preparation	68 (46.3)	36 (27.9)	72 (47.4)	53 (39.6)	0.45 (0.20; 1.05)	0.47 (0.19; 1.13)	0.07 (-0.03; 0.17)
	Action/ Maintanance	79 (53.7)	93 (72.1)	80 (52.6)	81 (60.5)			
Smoking Secession	Precontemplation/ Contemplation/ Preparation	34 (34.3)	27 (31.8)	25 (27.2)	18 (23.7)	2.08 (0.23; 18.6)	1.83 (0.20;16.4)	0.00 (-;-)
	Action/ Maintanance	65 (65.7)	58 (68.2)	67 (72.8)	58 (76.3)			

Data are n (%). ICC = Intracluster correlation coefficient, MI: Mixed models conducted after across cluster multiple imputation of missing values (sensitivity analysis)

Statistical analysis used mixed models including a random intercept to adjust for cluster effect, the respective baseline value, and covariates age, sex and smoking status of participants at baseline, and the variables used for minimization [sex and medical specialization of PCP, further training in diabetology and the socio-economic environment of the practices].

Fig. 1: Impact of times of hard lockdown due to coronavirus SARS-CoV-2 pandemic on mean physical activity

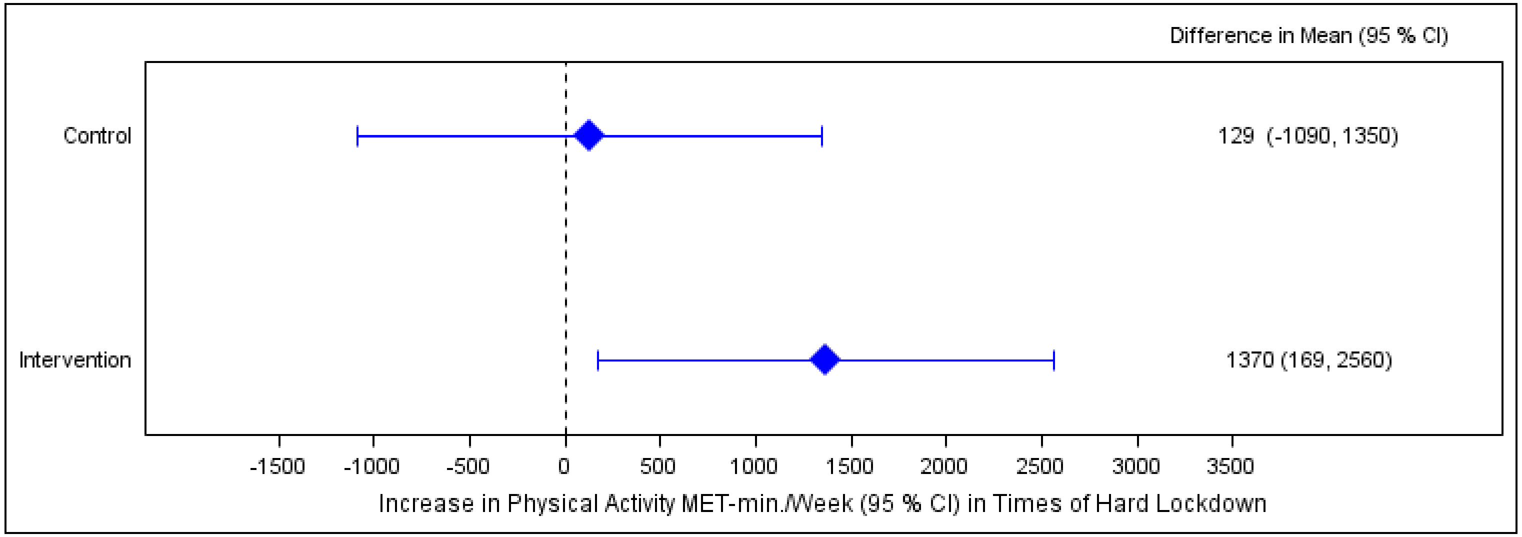

Supplement: Supplementary file 1 — Supplementary file1 (PDF 85 kb) [file 592_2022_1895_MOESM1_ESM.pdf]
